# Annual Meeting of the International Dental Publication Company

**Published:** 1892-03

**Authors:** 


### ANNUAL MEETING OF THE INTERNATIONAL DENTAL PUBLICATION COMPANY.

The Tenth Annual Meeting of this Company was held at Jersey City, January 23, at which a large majority of the stock was represented.

The usual routine business was transacted of reading the reports, auditing accounts, and the election of officers, of which the old list was retained with the exception of Dr. Jacob L. Williams, of Boston, who declined re-election. His place was filled by the election of Dr. Horatio C. Meriam, of Salem, Mass.

The meeting was marked by an earnest purpose to foster the interests of the INTERNATIONAL DENTAL JOURNAL. In connection with this an important address was made by Dr. J. Morgan Howe in reference to the obligation of the members of the company to give their strength to this periodical by so placing their papers that they will be assured of publication in the INTERNATIONAL DENTAL JOURNAL. The reports presented exhibited a year of very satisfactory work in the business relations, and it was also apparent that the profession were beginning to realize the importance of sustaining a periodical devoted exclusively to its interests.

